# Insights into Cancer Immunotherapies: Recent Breakthroughs, Opportunities, and Challenges

**DOI:** 10.3390/cancers15041322

**Published:** 2023-02-19

**Authors:** Evan G. Pappas, Michael H. Kershaw, Clare Y. Slaney

**Affiliations:** 1 Cancer Immunology Program, Peter MacCallum Cancer Centre, Melbourne, VIC 3000, Australia; 2 Sir Peter MacCallum Department of Oncology, University of Melbourne, Parkville, VIC 3010, Australia

This Special Issue reminds us that, although incredible developments have occurred in the field of cancer immunotherapy, there is still plenty of room for improvement. Although many otherwise untreatable patients have benefited from novel therapies, we still do not completely understand why some patients respond while others do not. Here, experts in the field summarize recent breakthroughs, but they also examine the challenges and opportunities in improving current therapies. These discussions are increasingly important because cancer immunotherapy has become a focal point in the race toward a cure.

Many manuscripts from this Special Issue focus on improving and better understanding current immunotherapies. For example, Lau and colleagues lay the groundwork for enhancing the clinical efficacy of adoptive cell transfers [1], and others identify novel antigens [2]. An innovative approach by Jazowiecka-Rakus and collaborators boosted the effectiveness of oncolytic viruses [3], while Ponath et al. redeployed histone deacetylase inhibitors to improve natural killer cell immunotherapy [4]. Such advancements rely on understanding existing therapies to identify suitable opportunities. This is exemplified by the work of Wu et al., who revealed avenues for improving chimeric antigen receptor (CAR) T cells by elucidating how they differ from conventional T cells [5]. In addition to iterative improvements, Maruoka and colleagues also embraced new technologies by leveraging recently developed photoimmunotherapy [6]. Together, these studies pave the way for future clinical advancement.

Once a treatment completes clinical trials, it must continue to be scrutinized for efficacy and safety. Notably, Choi and colleagues studied an immune checkpoint inhibitor (ICI) that may be less effective than previously reported [7]. They demonstrated the importance of retrospectively studying real-life clinical experiences beyond trials. An opinion piece featured here also discusses current knowledge in the field of ICIs and how they can be leveraged to improve clinical outcomes for patients with HER2-positive breast cancer [8]. These articles promote the idea that the bench-to-bedside process is indeed circular and not linear.

In recent years, a wealth of knowledge has been generated and many of the included reviews focus on the key cellular players underpinning tumor immunology and the cytokines that control them. Examples include the importance of understanding metabolism in CAR T cells [9] and not minimizing the physical properties of the CARs and their direct impact on clinical outcomes [10]. Similarly, the use of CARs in a variety of other cell types beyond T cells is reviewed [11], reminding us that the pursuit of innovation is within reach. While cells play a central role in immunotherapies, we must not underestimate the complex contribution of the cytokines that influence them. For example, the interleukin 12 family of cytokines, and their roles in both improving and suppressing immune responses, are discussed in depth [12]. Similarly, a pleiotropic cytokine, tumor necrosis factor alpha, is also implicated in many processes that can promote or hinder effective anti-cancer responses [13]. Such reviews help demystify complicated cellular interactions, in turn informing better future therapies.

The potential of non-T cells in immunotherapy is increasingly appreciated, including natural killer cells [14] and dendritic cells [14]. The success of these strategies relies on the immune recognition of cancer cells. It is imperative that we better understand the mechanisms involved in the frequent failure to recognize tumor antigens [15] and the evasion of immune responses [16]. 

Emerging strategies to modulate the immune system are also dissected in this issue. For instance, the use of nonreplicating adenovirus for gene therapy is explored [17], while another manuscript addressed the opportunity of targeting dynamic kinases expressed on both immune and tumor cells [18]. New immunotherapies can be associated with toxicity and, thus, clinical tolerance was discussed in some reports included in this issue. For example, the use of pattern recognition receptors (PRR) to improve immune responses must be balanced against potential harm and side effects. Some intratumorally administered PRR agonists have recently been investigated, and they possess more favorable safety profiles [19]. Complications have also been reported with the use of other immunotherapies, such as anti-CD3-bispecific antibodies [20]. Reviewed by Middelburg et al., anti-CD3-bispecific antibody therapies, like CAR T cells, are therapeutically effective in hematological cancers, but they fail to treat solid tumors [21]. Hurdles include the immunosuppressive tumor microenvironment and the difficulty for T cells to infiltrate the tumors [22]. Another innovative strategy involves the use of nanoparticles and, more recently, combining them with immunotherapies [23].

Glioblastoma, an aggressive tumor of the brain, is characterized by poor clinical outcomes. Many animal studies failed to capture key human disease features, and the results were not successfully translated clinically. One article from this issue discusses the complexity of conducting and interpreting clinical trials featuring immunotherapies for glioblastomas [24], while another article focuses on key limitations in the animal models informing these trials [25].

Overall, the field of cancer immunotherapy represents an exciting breakthrough. Improved understanding has resulted in novel therapeutics, which have, in turn, been optimized. Despite such impressive advancements, many patients either lack access to or fail to benefit from treatment. Future work must improve accessibility, as well as stratify patients to better address their individual needs. This will allow a golden age of personalized immunotherapy in oncology.
